# Changing Health Beliefs about Breast Cancer Screening among Women in Multi-Ethnic Malaysia

**DOI:** 10.3390/ijerph19031618

**Published:** 2022-01-30

**Authors:** Mila Nu Nu Htay, Maznah Dahlui, Désirée Schliemann, Christopher R. Cardwell, Siew Yim Loh, Nor Saleha Binti Ibrahim Tamin, Saunthari Somasundaram, Michael Donnelly, Tin Tin Su

**Affiliations:** 1 Centre of Population Health, Department of Social and Preventive Medicine, Faculty of Medicine, University of Malaya, Kuala Lumpur 50603, Malaysia; drmlnnh@gmail.com; 2 Department of Community Medicine, Manipal University College Malaysia, Manipal Academy of Higher Education (MAHE), Melaka 75150, Malaysia; 3 Department of Health Policy and Administration, Faculty of Public Health, Universitas Airlangga, Surabaya 60115, Indonesia; 4 Centre for Public Health and UKCRC, Centre of Excellence for Public Health, Queen’s University Belfast, Belfast BT12 6BA, UK; D.Schliemann@qub.ac.uk (D.S.); c.cardwell@qub.ac.uk (C.R.C.); michael.donnelly@qub.ac.uk (M.D.); 5 Department of Rehabilitation Medicine, Faculty of Medicine, University of Malaya, Kuala Lumpur 50603, Malaysia; syloh@um.edu.my; 6 Ministry of Health, Putrajaya 62590, Malaysia; drnorsaleha@moh.gov.my; 7 National Cancer Society, Kuala Lumpur 50300, Malaysia; saunthari@gmail.com; 8 South East Asia Community Observatory (SEACO) and Global Public Health, Jeffery Cheah School of Medicine and Health Sciences, Monash University Malaysia, Bandar Sunway 47500, Malaysia

**Keywords:** breast cancer, mass media campaign, mammogram, screening, health beliefs

## Abstract

This study evaluated the impact of the ‘Be Cancer Alert’ mass media campaign for breast cancer (BCAC-BC) in terms of changes to women’s health beliefs regarding BC susceptibility and the benefits and barriers of breast cancer screening in Malaysia. Pre- and post-campaign surveys evaluated changes in health beliefs among women aged 40 years and above (*n* = 676). The perceived susceptibility to breast cancer was significantly higher at follow-up (mean ± SD: 7.30 ± 2.77 vs. 7.63 ± 2.58, *p* = 0.008) whereas the mean score for the perceived benefits of undertaking screening was high at baseline and follow-up (16.34 ± 2.36 vs. 15.95 ± 2.07, *p* = 0.001). The perceptions or beliefs about barriers to screening did not change significantly (31.70 ± 8.26 vs. 31.77 ± 7.63, *p* = 0.841). Regression analyses indicated that mean scores for the barriers subscale were significantly lower among Chinese women (−2.61, 95% CI −4.67, −0.55, *p* = 0.013) compared to Malay, and among single compared to married women (−2.40, 95% CI −4.60, −0.21, *p* = 0.032) after adjustment for other demographic variables and past screening history. Malaysian women appeared to already have positive perceptions before the BCAC-BC mass media campaign about the benefits of BC screening. However, the campaign appeared to be linked to both an increased awareness of the susceptibility to breast cancer and to positive beliefs that countered emotional barriers to screening, particularly among single women and Chinese-Malay women.

## 1. Introduction

Breast cancer is a global health challenge and the most common cancer among females worldwide [1]. The incidence of breast cancer is increasing in Asia. For example, in Malaysia, the age-standardized rate (ASR) of breast cancer was 31.1 in 2007–2011 [2] compared to 34.1 per 100,000 women in 2012–2016 [3]. The Malaysian Ministry of Health (MoH) recommends annual clinical breast examination (CBE) for women who are 40 years and older and annual or biennial mammogram screening for women who meet specified criteria [4]. A woman who has at least one of the following criteria is recommended to have an annual mammogram: (i) a significant family history, i.e., first-degree family with breast cancer (mother, sister, daughter), (ii) a carrier of BRCA1 and two genetic mutations, or (iii) atypical hyperplasia in breast biopsy. A biennial mammogram is recommended for women who have at least two of the following criteria: (i) never gave birth (or) gave birth to a child after 30 years of age, (ii) early menarche (less than 12 years of age), (iii) late menopause (more than 55 years of age), (iv) receiving hormone replacement therapy (HRT), and (v) obesity: body mass index (BMI) ≥ 27.5 [4].

Early detection is essential to improve survival, and screening interventions have been proven to detect breast cancer at an early stage. Clinical breast examination is an important mode of screening for the early detection of breast cancer, especially in low- and middle-income countries. For example, a recent longitudinal study in India reported that CBE significantly reduced late-stage diagnosis and that there was a 30% relative reduction in BC mortality among women 50 years and above [5]. CBE is provided at primary health clinics, whereas a mammogram is available only at certain government hospitals, private healthcare facilities, and via subsidized programs [4,6,7,8]. A subsidized mammogram screening was initiated in 2007, though subsidized screening centers tend to be concentrated in urban areas [9]. An effective screening program depends on uptake and adherence [10]. In Malaysia, the uptake of screening is low to moderate—approximately 65% of women have had a CBE in their lifetime [6], 21% have received a mammogram within the last three years, and 63% of mammograms were provided in government or public healthcare facilities, whereas just over one third occurred in the private sector [11].

Health-awareness-raising campaigns intend to transform the attitudes and behaviors of a targeted community and to change traditional and social norms [12]. Campaigns or programs that are theoretically informed tend to have a greater likelihood of exerting impact in these domains [12]. For example, the Health Belief Model (HBM) is an early theory of behavior change that has been used widely to design and inform health-related campaigns and health promotion interventions and to predict the behaviors of given populations [13,14,15]. Attitudes, beliefs, and perceptions tend to be linked to individual behaviors, including the uptake (or lack thereof) of screening and prevention programs [16,17]. For example, the Champion Health Belief Model Scale (CHBMS) posits that a women’s perceptions about her susceptibility to breast cancer, the benefits that she thinks that she would derive from screening, and the barriers to the utilization of screening that she thinks that she would encounter will influence her uptake of mammogram screening [18]. The concept of perceived susceptibility and perceived severity are related to a threat that could predict the behaviors of people. Regarding breast cancer screening, women must recognize their perceived susceptibility to breast cancer as a threat in order to uptake the screening [18]. Furthermore, some studies have found that breast cancer awareness activities and programs that were informed by, or revolved around, beliefs and the Health Belief Model (HBM) reduced erroneous beliefs and dispelled myths about breast cancer and mammograms [19], improved health beliefs about breast cancer screening [20], and increased the mammogram screening uptake [21]. Previously conducted mass media campaigns in high-income countries such as the USA and UK have been associated with an increased mammogram uptake during the campaign period [22] and an improved awareness about risk factors for breast cancer (e.g., alcohol) [23]. Therefore, with the MoH and the National Cancer Society of Malaysia, we co-designed a culturally acceptable, theoretically informed, and evidence-based mass media campaign called the Be Cancer Alert Campaign (BCAC) [24,25]. This paper reports the results of an evaluation that assessed the possible impact of the BCAC in terms of changes in the health beliefs held by Malaysian women about breast cancer screening.

## 2. Materials and Methods

The Be Cancer Alert Campaign occurred between September and October 2018 for a five-week duration. The protocol for the evaluation and details about the cultural adaptation of the BCAC were published previously [24] and explained briefly here. The Research Ethics Committee, University of Malaya (ID: 2016126–4668), and the National Medical Research Register (ID: NMRR-18-1961-42562) granted ethical approval.

### 2.1. Study Design, Sample Size, and Sampling Method

A population-based, pre-, and post-intervention study was conducted in Petaling District, Selangor State, Peninsular Malaysia. Petaling District consists of mainly urban and sub-urban areas and its population is reflective of the national population and contains a mix of ethnicities, age groups, and income groups. The sampling unit comprised households in the study area that were selected randomly by the Department of Statistics Malaysia (the sampling frame was unavailable to the research team). Respondents were recruited from the randomly selected households in the study area. The inclusion criteria were women aged 40 years and above living in the randomly selected households who spoke Malay or English and were able to complete questionnaires independently. Respondents with cognitive impairment were excluded. Written informed consent was obtained from eligible women after each woman was informed about the study, including aims, data collection, and procedures. Women who participated in the pre-intervention survey were invited to participate in the post-intervention survey.

The sample size for our pre-and post-intervention study design was based on a standard deviation of the within-pair difference of 5.06 for perceived barriers subscale from a previous study [17], allowing us over 80% power to detect, as statistically significant, a true mean difference of 0.61 in pre- versus post-barriers subscale; this was similar to that seen in a previous study [17].

The estimated sample size (n) was calculated by using the following formula:

$$n={\left(\frac{z_{1-\propto }-z_{\beta }}{\delta }\right)}^{2}$$

where *α* = significant level, *β* = probability of a type II error, and *δ* = effect size [26].

The minimum sample size for our pre- and post-intervention study design was estimated to be 541 women.

### 2.2. Mass Media Campaign

The Be Cancer Alert Campaign (BCAC) was grounded in the theoretical framework of the Health Belief Model. The campaign materials were adopted from the Be Cancer Aware campaign in Northern Ireland (with permission from the Northern Ireland Public Health Agency). Two expert panel discussions were conducted with 3 family physicians, 4 public health specialists, 1 language and linguistic expert, 4 cancer advocates, and 5 media and communication experts to culturally adapt the campaign materials. The campaign materials were translated into local languages and adapted to ensure that they were culturally acceptable for the various contexts, traditions, and beliefs in Malaysia [25].

During the adaptation process, we conducted a total of six focus groups (two FGs per ethnic community) with men and women who earned a low to middle income. Each FG comprised 10 participants/respondents. The findings of the FG discussions were used to inform, refine, and adapt the campaign messages, and to prepare for their delivery through various media modes and channels. For example, the FG findings indicated that campaign information and messages were likely to have a stronger resonance if they were delivered by cancer survivors and/or via a positively perceived celebrity personality rather than by healthcare personnel. According to the collective views of FG participants, there was a need to ensure that the materials and designs would be eye-catching and that the messages would positively encourage women. Importantly, respondents suggested highlighting the valuable role and function of family and particular family members In screening and managing cancer. The campaign contained content and delivered messages about symptoms, risk factors, screening programs, and the benefits of screening and early detection. The nationwide BCAC-BC was delivered through television, radio, social media, and websites, whereas print media (billboards, banners, posters, and brochures) were distributed in the local Petaling District. Further details on the implementation of the BCAC-BC mass media campaign are presented below.

#### 2.2.1. Television and Radio

A 30 s TV advertisement about breast cancer awareness in Malay, English, and Chinese was aired nationwide for four weeks. In addition, radio stations were used to deliver key messages in 30 s ‘slots’ about breast cancer for two weeks. The two radio channels that aired campaign messages used English and Tamil languages only.

#### 2.2.2. Print Media

Print materials were prepared in four languages—Bahasa Malaysia (BM), English (ENG), Mandarin (MAN), and Tamil (TAM)—and were implemented in the form of billboards (BM), buntings (BM), posters (BM and ENG), banners (BM and ENG), and brochures (BM, ENG, MAN, TAM) that were displayed at the roadsides and distributed to supermarkets and (public and private) health care clinics in Petaling District.

#### 2.2.3. Social Media and Website

Online information about breast cancer and screening was delivered through the bespoke BCAC website (Available online: http://www.becanceralert.com) (accessed on 30 January 2022) and the website and Facebook page of the National Cancer Society Malaysia (NCSM). In addition, the National Cancer Society toll-free helpline catered for requests for further information and inquiries from the community.

### 2.3. Assessment of Beliefs

Socio-demographic information and beliefs about breast cancer screening were collected as part of a face-to-face interview with study respondents. Trained enumerators collected the data from study respondents who provided informed consent. Data collection was carried out three months before and after the campaign. Past history of breast cancer screening was recorded in terms of whether or not a woman had had a CBE within the last year or had had a mammogram within the last two years. Previous screening uptake history was collected and recorded during the research interview with each respondent. The revised version of CHBMS from the US [18] was translated into Malay language and validated to assess the health beliefs of women about breast cancer and screening [27]. Malay is the official national language of Malaysia and respondents were able to understand and answer the Malay version of the questionnaire—the CHBM-BC-M. The CHBMS-BC-M includes three subscales: perceived susceptibility (3 items), perceived benefits (4 items), and perceived barriers (14 items). Responses were recorded on a five-point Likert scale with the following scoring: strongly disagree = 1, disagree 2, undecided = 3, agree = 4, strongly agree = 5. The internal consistency or reliability of the CHBM-BC-M as indicated by Cronbach’s alpha was 0.92, 0.59, and 0.78, respectively, for susceptibility, benefits, and barriers subscales.

### 2.4. Data Analysis

Descriptive statistics were used to describe the socio-demographic characteristics of respondents. Individual item mean scores at baseline and follow-up were compared using paired sample *t*-tests. Total subscale scores were calculated: susceptibility subscale (range 3–15), benefits subscale (range 4–20), and barriers subscales (range 14–70). Theoretically, we interpreted a higher awareness about the possible risk of getting breast cancer and a higher score for the perceived benefits of screening subscale as indicating positive improvements in health beliefs. Conversely, a lower perceived barriers subscale score was considered to indicate an improvement in health beliefs regarding breast cancer screening [17,18]. The mean score of each subscale at baseline and follow-up was compared using a paired sample *t*-test. Multiple linear regression analysis investigated the association between respondents’ characteristics and changes in health beliefs for each subscale. The total subscale score of susceptibility, benefits, and barriers, respectively, were conceptualized as dependent variables. Sociodemographic variables expressed recognition (or not) of the BCAC-BC campaign, and previous screening uptake history weas entered as an independent variable. Missing values in our data across all variables were less than 5%. According to previous research, a proportion of missing values below 5% is inconsequential for the findings of a study [28,29]. Therefore, we proceeded to undertake data analysis using the full data set without imputation. Data were analyzed using PASW Statistics for Windows, Version 18.0 (SPSS Inc., Chicago, IL, USA).

## 3. Results

During the baseline survey, a total of 992 women responded, and in the post-campaign survey, 676 (68%) women responded to the interview. The analysis presented in this paper is based on respondents who completed baseline and follow-up assessment measures (*n* = 676). The characteristics of (i) all respondents at baseline, that is, who participated in the pre-intervention or pre-campaign survey, (ii) respondents who participated in the pre-intervention survey only, and (iii) respondents who participated in both pre- and post-intervention surveys are reported in Table 1.

Approximately 41% of respondents were between 40–49 years of age and 30% were between 50–59 years of age. The age distribution is similar to the Malaysia population age distribution among women, except for the 40–49 years age group (approximately 36% in the general population vs. 41% in the study sample) [30]. The majority of respondents (93%) were Malaysians (Malay, 51.6%; Chinese, 22.3%; Indian, 17.8%), married (86.8%), and not in paid employment (75.6%). Approximately half had completed secondary education (54.2%). Most respondents (71.2%) had a monthly family income of less than RM 4000. One-third (34.6%) of respondents reported that they had received CBE within one year and 26% had a mammogram in the last two years.

Table 2 compares CHBMS-BC-M item and subscale mean scores of pre- and post-campaign surveys. Overall, awareness of the risk of breast cancer increased slightly after the campaign (7.30 vs. 7.63, *p* = 0.008). Responses indicated disagreement with each statement or item about perceived susceptibility (scoring less than three at baseline and follow-up surveys). In the benefits subscale, mean item scores were above three, indicating agreement with the statements at baseline and follow-up surveys.

Regarding the barriers subscale, mean item scores of 3 and below were observed for all statements at both survey time points, thereby indicating disagreement. A significant mean score reduction was observed for four (of the fourteen) items: “I am afraid to have breast cancer screening because I might find out something is wrong” (2.53 ± 1.32 vs. 2.35 ± 1.21, *p* = 0.004), “I am afraid to have breast cancer screening because I don’t understand what will be done” (2.36 ± 1.27 vs. 2.16 ± 1.15, *p* < 0.001), “I don’t have the encouragement I need from my close relatives to attend breast cancer screening” (2.14 ± 1.17 vs. 1.98 ± 0.98, *p* = 0.004), and “I don’t know how to go about getting breast cancer screening” (2.34 ± 1.30 vs. 2.03 ± 1.05, *p* < 0.001).

The respondents’ concerns about mammogram screening increased: “Having breast cancer screening (mammogram) is too painful” (2.85 ± 1.19 vs. 3.00 ± 1.07, *p* = 0.004), and “Having breast cancer screening (mammogram) exposes me to unnecessary radiation” (2.58 ± 1.08 vs. 2.86 ± 0.88, *p* < 0.001) (Table 2). Women who had had previous experience of a CBE or a mammogram perceived fewer barriers regarding concerns about pain and radiation exposure compared to women without screening experience (CBE: 2.43 vs. 2.66, *p* = 0.007) (mammogram: 2.36 vs. 2.66, *p* = 0.002) (Supplementary Table S1). The summed 14-item perceived barriers scale was significantly lower in women who had a history of CBE and mammogram use compared to women who did not report a screening uptake history (Table 3).

In the regression model, the dependent variables were the total scores for the subscale that measured susceptibility, benefits, and barriers, respectively. The independent variables were nationality, ethnicity, age, marital status, education, monthly income, employment status, previous screening history (CBE and mammogram), and BCAC-BC campaign recognition. Normality tests indicated that the total scores for the subscales (susceptibility, benefits, and barriers) were normally distributed. The VIF value was less than 1.23 for all independent variables, thereby indicating little or no collinearity between independent variables. The residuals were not normally distributed for the subscales of the CHBMS-BC-M, and multiple regression analysis was used to identify which factors were associated with the subscales. A reduction in perceived barriers was associated with ethnicity (Chinese −2.61, 95% CI −4.67, −0.55, *p* = 0.013) and marital status (single −2.40, 95% CI −4.60, −0.21, *p* = 0.032). Socio-demographic variables were not associated with a change in the subscale scores of the perceived susceptibility and benefits (Table 4).

## 4. Discussion

This is the first study to evaluate the impact of a mass media campaign on women’s health beliefs about breast cancer screening in Malaysia. Theoretically, women’s perceived susceptibility to breast cancer risk is likely to improve the uptake of breast cancer screening [18]. The three statements or items that comprise the susceptibility subscale enquired about a woman’s beliefs regarding the extent to which she perceived that she was at risk and vulnerable to experiencing breast cancer; a higher score indicated a higher agreement with the statements and a higher level of susceptibility. On average, the women in our study had a mid-level degree of perceived susceptibility to breast cancer in their lifetime. A similar finding was observed in a peer education intervention study that aimed to improve breast cancer awareness and screening in Turkey [31]. Previous studies have reported an association between susceptibility and the uptake of CBE [32] and a mammogram screening [33] and also found that susceptibility predicted future mammogram screening intention in the United States [34]. Our study indicated that women had an increased level of perceived susceptibility following the completion of the BCAC-BC campaign. A similar health belief change was observed for female students in a study conducted in Egypt after an educational video intervention [35], though not in a study of female teachers in northern Turkey, in which women’s perceived susceptibility to breast cancer appeared to decrease after a video intervention (8.29 ± 1.83 vs. 7.48 ± 1.78) [17]. It is important that cancer-awareness-raising programs appropriately raise the level of susceptibility in terms of improving awareness about BC risk while guarding against making women feel fearful or apprehensive about their breast health. Previous mass media campaigns or interventions in the US [22], UK [36], and Korea [19] reported improvements in symptom awareness, screening uptake, referral rate, and a reduction in myths about breast cancer. However, this study presents the first evaluation of changes in health beliefs in Malaysia as indicators of the impact of a breast health promotion mass media campaign (the BCAC-BC). A mid-level point, approximately, on a scaled response seems to indicate that the presentation of the program campaign messages attained an appropriate balance. The perceived susceptibility did not differ between women with a history of screening uptake and women without screening experience. It is possible that the intervention campaign/program ‘levelled up’ any differences in awareness and susceptibility. The extent to which a change in beliefs about susceptibility translates to an increased breast screening uptake requires a longer-term evaluation.

It is reasonable, all other things being equal, to posit that an understanding of the benefits of breast cancer screening is likely to lead to positive perception about screening uptake, early detection, and treatment, and a better survival for breast cancer patients [18]. Interventions, such as peer education and teaching sessions, increase the perceived benefits of breast cancer screening [31,37], and mass media campaigns [38] appear to improve breast cancer awareness and knowledge. The mean average score in our study for the perceived benefits subscale did not increase or improve at the post-mass media campaign assessment point, perhaps because there were already high scores for three of the four items, pre- and post-assessment. The score for one item was lower than the score for each other item, but it was still in the range, indicating agreement (at baseline and follow-up). Generally, the Malaysian women in our study already had positive beliefs or perceptions about the benefits of BC screening before they saw the campaign.

Generally, perceived barriers to screening were less salient at follow-up, except for the two specific barriers (see below), and the scores were significantly reduced for four barriers after the campaign program compared to the baseline. These four items included emotion-related barriers, such as fear of finding out that something (about their breast health) was wrong, fear of attending screening because of their limited understanding about what they would be required to do, and a lack of encouragement and support from relatives to attend the screening, and a service-related barrier about not knowing how to access and use screening. In a related report, 40% of respondents stated that they discussed the BCAC-BC campaign with friends and family and 21.7% reported that they or their family or friends visited a doctor after seeing the campaign advertisement [39].

It appears that the mass media campaign advertisements reduced these kinds of barriers and improved the understanding of how to access screening similar to findings elsewhere [19].

The two specific barriers that were the exception to this generally positive pattern of results were mammogram-related perceived barriers regarding pain and radiation side effects. Each barrier increased after the campaign, though not for women who had had previous experience of BC screening; these women did not tend to hold fears about radiation in relation to the use of mammogram screening. The previous history of utilizing CBE and mammogram screening, respectively, was associated with lower scores for perceived barriers in the analysis of baseline data (manuscript under preparation). Similarly, a US study found that women who had experience of mammogram screening (compared to women with no screening experience of this kind) reported fewer barriers (e.g., cost and misconception that screening was “unnecessary”) [40]. A lower availability and underutilization of mammograms by respondents (26%) might limit awareness of mammography and might contribute, in part, to increasing perceived fears about pain and radiation among Malaysian women. There appears to be a need for BC screening education that focuses on misbeliefs about the side effects of mammograms, such as intolerable pain and radiation, and that clearly communicates the benefit of BC screening. Primary care physicians play an important role in explaining BC risks, susceptibility, the benefit of the screening, and misperception in order to reduce the barriers of BC screening.

The results of the analysis indicated ethnic group variation regarding health perceptions and beliefs. Chinese women compared to Malay women appeared to gain more from the campaign in terms of perceiving fewer barriers to the uptake of breast cancer screening. Since this is the first evaluation of a mass media campaign to raise breast cancer awareness and investigate health beliefs in Malaysia, there are no comparative studies on the variation between ethnic communities or groups. Our study findings revealed that cancer awareness-raising programs have the potential to reduce perceived barriers and improve the mammogram screening uptake among women in Malaysia. Future programs and interventions need to take account of the need to target particular ethnic groups, such as Malay and ‘other’ ethnic women groups. It is important, too, to be mindful of existing differences between ethnic groups in Malaysia, such as the income status [41], employment [41], cultural values [42], religious beliefs [42], and healthcare-seeking [43], and how these differences tend to intersect and intertwine with a woman’s health beliefs. During the follow-up survey, attrition was significantly higher among the Chinese women, as well as among elderly women and employed women. A lack of interest in the program and cultural mistrust were reported as possible explanatory factors for attrition among ethnic groups in a US health promotion trial [44]. The reasons for attrition in our study are unclear and may be related to inconvenience due to work commitments and perhaps lost interest among elderly women.

It is important to recognize that it was not possible to employ an experimental evaluation design, and, at best, women might be described only in terms of serving as their own ‘control’ in relation to pre-post program campaign assessments. In Malaysia, the television and radio channels are transmitting nationwide, and there is no local television or radio channel. Therefore, our BCAC-BC campaign reach was nationwide. In the absence of a formal control group of women who were not exposed to a mass media campaign (a noted study limitation), we used responses to questions to construct a quasi-comparator group (e.g., reported recognition vs. no recognition). Previous studies have adopted a similar analytical strategy in this circumstance; for example, a study of an anti-smoking campaign in the USA [45]. The translation of women’s health beliefs into action in the form of an improved BC screening uptake merits consideration. Mass media campaigns may serve as a kind of cue or cues to action regarding screening uptake [46], provided, of course, that screening services are available and accessible [9]. An improvement in breast cancer symptom awareness, an increased sense of the risk of breast cancer, and a reduction in emotional barriers related to the uptake of screening were observed after the BCAC-BC mass media intervention [39]. The perceived benefits were already close to ceiling-high before the campaign and remained high at follow-up. Generally, there was a low level of perceived barriers to screening. Fear of mammogram screening, specifically, appeared to increase post-campaign, though the endorsement of response values remained within a range that did not point, overall, to a potentially serious barrier. Health beliefs might take a longer period to noticeably change, and the repeated implementation of the campaign with tailored messages about, for example, the benefits and misbeliefs regarding the side effect of mammogram screening targeted towards particular ethnic groups, such as Malay women, merit consideration by public health service planners.

## 5. Limitations

The study was conducted in the Petaling District, which consists of mainly urban areas, and, therefore, the findings might not be generalizable to the rural community. Ideally, a comparator area or one or more control groups would have provided a stronger test of the impact of the intervention. However, due to the nationwide reach of the mass media campaign (TV and radio), it was not feasible to construct control regions within the country that were not exposed to the media campaign. Therefore, the pre- and post- interventional comparison was conducted as a pragmatic approach to evaluate the impact of the BCAC-BC campaign. The CHBMS-BC-M used as a study instrument was translated and validated from the original English version, which did not include the severity subscale. Therefore, women’s perceived severity of breast cancer was not able to be assessed in this study. A further possible limitation to note may be self-reported bias. For example, women’s previous CBE and mammogram uptake history data were collected based on their verbal responses to interview questions and we were unable to countercheck their responses with their health records. The evaluation was conducted over a short-term period, and, therefore, it was not possible to appraise the long-term effects on the health beliefs and screening uptake. The non-completion of the post-intervention survey by some Chinese women, women 70 years and older, and employed women may limit the generalizability of the findings of these subgroups. The BCAC-BC mass media campaign was conducted in October, ‘breast cancer month’, and it is possible that some respondents were exposed to other breast cancer awareness activities during that month, though the research interview investigated and analyzed respondent exposure to BCAC-BC specifically.

## 6. Conclusions

In conclusion, the findings of the study indicated that women’s perceived susceptibility to breast cancer during their lifetime increased following participation in the BCAC-BC campaign. Furthermore, perceptions about barriers, particularly emotional barriers, to the screening uptake reduced significantly after the campaign intervention. The women in our study had positive perceptions about the benefits of BC screening before they saw the campaign, and the lack of significant pre-post campaign differences may reflect a ceiling effect. There is a need for BC health promotion programs to take account of the finding that health beliefs or perceptions about BC and screening appear to vary across ethnic groups. Finally, cancer-awareness-raising media programs appear to reduce perceived barriers and improve the mammogram screening uptake among women in Malaysia, but the extent to which change in beliefs translates to a sustained increased breast screening uptake requires a longer-term evaluation.

## Figures and Tables

**Table 1:** Sociodemographic characteristics of respondents.

Variable	All Survey Participants at Pre-Intervention or Baseline (*n* = 992)	Pre-Intervention Survey Participants Only (*n* = 316)	Pre- and Post-Intervention Survey Participants (*n* = 676)	*p* ^1^
	*n* (%)	*n* (%)	*n* (%)	
Nationality				
Malaysian	920 (92.7)	291 (92.1)	629 (93.0)	
Non-Malaysian	72 (7.3)	25 (7.9)	47 (7.0)	0.601
Ethnicity				
Malay	499 (50.3)	150 (47.5)	349 (51.6)	
Chinese	253 (25.5)	102 (32.3)	151 (22.3)	
Indian	159 (16.0)	39 (12.3)	120 (17.8)	
Others	81 (8.2)	25 (7.9)	56 (8.3)	0.004
Age				
40–49 years	378 (38.5)	104 (33.1)	274 (41.0)	
50–59 years	301 (30.7)	102 (32.5)	199 (29.8)	
60–69 years	204 (20.8)	67 (21.3)	137 (20.5)	
70 years and above	99 (10.1)	41 (13.1)	58 (8.7)	0.044
Age (mean ± SD)	54.56 (10.45)	55.81 (10.83)	53.98 (10.22)	
Marital Status				
Married	852 (85.9)	265 (83.9)	587 (86.8)	
Single ^2^	140 (14.1)	51 (16.1)	89 (13.2)	0.240
Educational level ^3^				
No formal education	133 (13.4)	45 (14.3)	88 (13.0)	
Primary education	149 (15.1)	52 (16.6)	97 (14.4)	
Secondary education	524 (53.0)	158 (50.3)	366 (54.2)	
Tertiary education	183 (18.5)	59 (18.8)	124 (18.4)	0.666
Monthly family income ^4^				
Below RM 4000	646 (69.8)	189 (66.5)	457 (71.2)	
RM 4001- RM 10,000	212 (22.9)	72 (25.4)	140 (21.8)	
RM 10,001 and above	68 (7.3)	23 (8.1)	45 (7.0)	0.366
Current Job Status ^5^				
Employed	269 (27.1)	104 (32.9)	165 (24.4)	
Unemployed	723 (72.9)	212 (67.1)	511 (75.6)	0.006
Clinical breast examination ^6^				
Yes	347 (35.1)	114 (36.3)	233 (34.6)	
No	641 (64.9)	200 (63.7)	441 (65.4)	0.617
Mammogram ^7^				
Yes	269 (27.2)	94 (29.7)	175 (26.0)	
No	720 (72.8)	222 (70.3)	498 (74.0)	0.221

^1^ Pearson chi-square [comparison between respondents who participated in the pre-intervention survey only (*n* = 316) and respondents who participated in both pre- and post-intervention surveys (*n* = 676)]. ^2^ Respondents who are widowed, divorced, and single. ^3^ No formal education—includes never schooled/never completed primary school; primary education—includes completed primary school; secondary education—includes completed form 3/completed form 5/certificate/A-level/STPM/HSC; tertiary education—includes diploma/bachelor degree/post-graduate degree. ^4^ Income of all household member combined. ^5^ Employed and/or studying—includes civil servant, private sector employee, self-employed, studying and working, still studying; Unemployed—includes government retiree, private retiree, homemaker, unemployed. ^6^ Self-reported CBE uptake within the last year. ^7^ Self-reported mammogram uptake within the last two years.

**Table 2:** Comparison of CHBMS-BC-M mean item and subscale scores pre- and post-BCAC-BC mass media campaign (*n* = 676).

CHBMS-BC-M		Pre-Intervention	Post-Intervention	*p* ^1^
		Mean (SD)	Mean (SD)	
Susceptibility				
1	It is likely that I will get breast cancer.	2.44 (1.00)	2.56 (0.96)	0.010
2	My chances of getting breast cancer in the next few years are high.	2.43 (1.00)	2.47 (0.89)	0.385
3	I feel I will get breast cancer sometime during my life. ^2^	2.43 (0.98)	2.61 (0.95)	<0.001
	Susceptibility subscale total score ^2^	7.30 (2.77)	7.63 (2.58)	0.008
Benefits				
1	If I get screened for breast cancer and nothing is found, I don’t need to worry much about breast cancer. ^3^	3.70 (1.21)	3.52 (1.12)	0.002
2	Having breast cancer screening will help me find breast lumps early.	4.28 (0.71)	4.23 (0.68)	0.149
3	Having breast cancer screening is the best way for me to find a very small lump.	4.25 (0.72)	4.19 (0.71)	0.168
4	Having breast cancer screening will decrease my chances of dying from breast cancer.	4.10 (0.82)	4.03 (0.79)	0.120
	Benefits subscale total score ^3^	16.34 (2.36)	15.95 (2.07)	0.001
Barriers				
1	I am afraid to have breast cancer screening because I might find out something is wrong. ^2^	2.53 (1.32)	2.35 (1.21)	0.004
2	I am afraid to have breast cancer screening because I don’t understand what will be done. ^2^	2.36 (1.27)	2.16 (1.15)	<0.001
3	I don’t know how to go about getting breast cancer screening. ^2^	2.34 (1.30)	2.03 (1.05)	<0.001
4	Having breast cancer screening is too embarrassing.	2.02 (1.09)	2.00 (1.05)	0.767
5	Having breast cancer screening takes too much time.	2.41 (1.13)	2.39 (1.06)	0.710
6	Having breast cancer screening (mammogram) is too painful. ^3^	2.85 (1.19)	3.00 (1.07)	0.004
7	People doing breast cancer screening are rough to women.	2.09 (0.87)	2.12 (0.90)	0.523
8	Having breast cancer screening (mammogram) exposes me to unnecessary radiation. ^3^	2.58 (1.08)	2.86 (0.88)	<0.001
9	I cannot remember to go to the doctor to get breast cancer screening.	2.05 (1.05)	2.11 (0.94)	0.210
10	I have other problems more important than getting breast cancer screening.	1.98 (0.99)	2.03 (0.90)	0.306
11	I am too not the right age to need a routine breast cancer screening.	2.06 (1.08)	2.14 (1.08)	0.157
12	I cannot afford to get breast cancer screening.	2.01 (1.01)	2.00 (0.87)	0.792
13	I don’t have the encouragement I need from my close relatives to attend breast cancer screening. ^2^	2.14 (1.17)	1.98 (0.98)	0.004
14	I am afraid that a male doctor will carry out the breast cancer screening.	2.75 (1.43)	2.79 (1.38)	0.556
	Barriers subscale total score	31.70 (8.26)	31.77 (7.63)	0.841

^1^ Paired sample *t*-test. ^2^ Positive direction/changes. ^3^ Negative direction/changes.

**Table 3:** Women’s health beliefs in relation to the past history of screening uptake (*n* = 676).

Health Beliefs	Women with History of CBE Uptake (*n* = 233)	Women without History of CBE Uptake (*n* = 441)		Women with History of Mammogram Uptake (*n* = 175)	Women without History of Mammogram Uptake (*n* = 498)	
	Mean (SD)	Mean (SD)	*p* ^1^	Mean (SD)	Mean (SD)	*p* ^1^
Susceptibility						
Pre-intervention	7.16 (2.85)	7.38 (2.72)	0.337	7.51 (2.97)	7.22 (2.69)	0.233
Post-intervention	7.63 (2.66)	7.65 (2.56)	0.920	7.91 (2.60)	7.55 (2.57)	0.114
Benefits						
Pre-intervention	16.11 (2.37)	16.46 (2.35)	0.066	16.52 (2.77)	16.27 (2.19)	0.224
Post-intervention	15.93 (2.00)	15.99 (2.12)	0.694	16.05 (1.97)	15.93 (2.11)	0.539
Barriers						
Pre-intervention	29.39 (8.16)	33.00 (8.02)	<0.001	28.12 (8.05)	33.05 (7.86)	<0.001
Post-intervention	30.43 (7.44)	32.79 (7.61)	<0.001	28.55 (7.27)	33.13 (7.36)	<0.001

^1^ Independent sample *t*-test.

**Table 4:** Multiple linear regression analyses of the relationship between socio-demographic characteristics and past experience of breast cancer screening and CHBMS-BC-M (*n* = 676).

Independent Variables		Susceptibility				Benefits						Barriers				
		Mean (SD) Difference between Pre- and Post-Intervention ^1^	B (Adjusted) ^2^	95% CI		*p*	Mean (SD) Difference between Pre- and Post-Intervention ^1^	B (Adjusted) ^2^	95% CI		*p*	Mean (SD) Difference between Pre- and Post-Intervention ^1^	B (Adjusted) ^2^	95% CI		*p*
				Lower	Upper				Lower	Upper				Lower	Upper	
Nationality																
Malaysian	629 (93.0)	0.33 (3.21)	Reference			−0.43 (3.01)		Reference				0.15 (8.98)	Reference			
Non-Malaysian	47 (7.0)	0.32 (3.61)	−0.02	−2.01	1.98	0.989	0.21 (2.65)	1.29	−0.44	3.01	0.144	−1.15 (8.79)	−5.09	−10.62	0.45	0.072
Ethnicity																
Malay	349 (51.6)	0.33 (3.10)	Reference			−0.25 (3.02)		Reference				0.72 (8.93)	Reference			
Chinese	151 (22.3)	0.49 (3.23)	0.21	−0.54	0.95	0.586	−0.29 (2.91)	0.33	−0.34	0.99	0.336	−1.17 (8.09)	−2.61	−4.67	−0.55	0.013
Indian	120 (17.8)	0.11 (3.57)	−0.56	−1.27	0.16	0.128	−1.06 (3.16)	−0.49	−1.14	0.15	0.135	0.16 (9.70)	−1.00	−2.98	0.97	0.320
Others	56 (8.3)	0.36 (3.42)	0.29	−1.61	2.19	0.766	−0.05 (2.55)	−0.60	−2.23	1.04	0.474	−0.61 (9.73)	3.57	−1.54	8.68	0.171
Age																
40–49 years	274 (41.0)	0.26 (3.25)	Reference			−0.42 (3.15)		Reference				0.55 (8.80)	Reference			
50–59 years	199 (29.8)	0.59 (3.15)	0.35	−0.29	1.00	0.281	−0.54 (2.97)	−0.03	−0.60	0.54	0.919	−0.45 (9.51)	−0.92	−2.72	0.87	0.313
60–69 years	137 (20.5)	0.14 (3.40)	−0.11	−0.89	0.66	0.773	−0.23 (2.83)	0.25	−0.44	0.94	0.476	−0.50 (8.78)	0.27	−1.88	2.42	0.803
70 years and above	58 (8.7)	0.31 (3.03)	−0.18	−1.25	0.89	0.740	−0.14 (2.79)	0.18	−0.79	1.15	0.719	0.95 (8.39)	2.67	−0.23	5.57	0.071
Marital Status																
Married	587 (86.8)	0.30 (3.20)	Reference			−0.38 (2.98)		Reference				0.24 (9.03)	Reference			
Single	89 (13.2)	0.53 (3.51)	0.31	−0.48	1.09	0.444	−0.38 (3.08)	−0.22	−0.92	0.48	0.541	−1.01 (8.53)	−2.40	−4.60	−0.21	0.032
Education																
No formal education	88 (13.0)	0.06 (3.22)	Reference			−0.16 (3.03)		Reference				−0.92 (8.65)	Reference			
Primary	97 (14.4)	0.49 (3.57)	0.64	−0.37	1.64	0.213	−0.55 (3.20)	−0.19	−1.08	0.70	0.673	−0.08 (10.43)	0.33	−2.47	3.13	0.817
Secondary	366 (54.2)	0.36 (3.12)	0.58	−0.27	1.44	0.180	−0.41 (2.88)	−0.01	−0.77	0.75	0.982	0.18 (8.80)	0.79	−1.62	3.19	0.522
Tertiary	124 (18.4)	0.32 (3.35)	0.73	−0.37	1.82	0.195	−0.31 (3.13)	0.41	−0.57	1.39	0.412	0.41 (8.48)	0.63	−2.44	3.69	0.689
Monthly family income																
Below RM 4000	457 (71.2)	0.44 (3.27)	Reference			−0.29 (3.02)		Reference				−0.02 (9.07)	Reference			
RM 4001–10,000	140 (21.8)	0.05 (3.09)	−0.57	−1.27	0.13	0.109	−0.25 (2.95)	0.01	−0.61	0.64	0.96	−0.05 (8.55)	−0.32	−2.27	1.62	0.746
RM 10,001 and above	45 (7.0)	0.24 (3.94)	−0.38	−1.49	0.74	0.510	−1.43 (2.48)	−1.53	−2.53	−0.52	0.00	1.49 (8.66)	1.85	−1.17	4.87	0.229
**Employment status**																
Unemployed	165 (24.4)	0.36 (3.18)	Reference			−0.33 (3.01)		Reference				−0.04 (9.16)	Reference			
Employed	511 (75.6)	0.25 (3.42)	−0.09	−0.73	0.56	0.795	−0.56 (2.93)	−0.16	−0.74	0.42	0.594	0.42 (8.39)	0.37	−1.42	2.16	0.685
**Past history of CBE** ^3^																
No	233 (34.6)	0.26 (3.29)	Reference			−0.48 (3.00)		Reference				−0.40 (8.55)	Reference			
Yes	441 (65.4)	0.46 (3.16)	0.28	−0.32	0.87	0.363	−0.18 (2.97)	0.54	0.00	1.07	0.048	1.00 (9.64)	1.11	−0.53	2.75	0.185
**Past history of Mammogram** ^4^																
No	175 (26.0)	0.32 (3.18)	Reference			−0.35 (2.90)		Reference				−0.06 (8.84)	Reference			
Yes	498 (74.0)	0.40 (3.41)	0.16	−0.50	0.83	0.629	−0.47 (3.21)	−0.10	−0.69	0.49	0.748	0.49 (9.38)	−0.24	−2.05	1.56	0.792
**BCAC-BC campaign**																
Non-recognizer	211 (31.2)	0.60 (3.31)	Reference			−0.43 (2.86)		Reference				−0.74 (9.39)	Reference			
Recognizer	441 (65.2)	0.18 (3.20)	−0.51	−1.10	0.07	0.086	−0.25 (2.99)	0.29	−0.23	0.81	0.276	0.07 (8.63)	−0.09	−1.72	1.55	0.919

^1^ SD—Standard deviation. ^2^ B (adjusted)—adjusted mean difference, ^3^ Respondents’ self-reported uptake of CBE within the last year. ^4^ Respondents’ self-reported uptake of mammogram within the last two years.

## Data Availability

The data presented in this study are available on request from the corresponding author.

## Supplementary Materials

The following supporting information can be downloaded at: https://www.mdpi.com/article/10.3390/ijerph19031618/s1, Table S1: Mammogram related barriers among women at pre- and post-intervention surveys (*n* = 676).
