# Supplementary material for: Changing Health Beliefs about Breast Cancer Screening among Women in Multi-Ethnic Malaysia
Source: Int J Environ Res Public Health. 2022 Jan 30;19(3):1618. doi: 10.3390/ijerph19031618 (PMC8835215; doi:10.3390/ijerph19031618)
Supplement: Supplementary file 1 [file ijerph-19-01618-s001.zip › ijerph-1481619-supplementary.pdf]

Supplementary Table

Table S1. Mammogram related barriers among women at pre- and post-intervention surveys (n=676)

**Mammogram related barriers**		**Women with history of CBE uptake**	**Women without history of CBE uptake**		**Women with history of mammogram uptake**	**Women without history of mammogram uptake**	
		Mean(SD)	Mean(SD)	p[1]	Mean(SD)	Mean(SD)	p[1]
Having breast cancer screening (mammogram) is too painful	Pre-intervention	2.85 (1.27)	2.85 (1.15)	0.949	2.73 (1.40)	2.89 (1.10)	0.121
	Post-intervention	2.99 (1.14)	3.00 (1.02)	0.900	2.92 (1.29)	3.02 (0.97)	0.275
Having breast cancer screening (mammogram) exposes me to unnecessary radiation.	Pre-intervention	2.43 (1.08)	2.66 (1.07)	0.007	2.36 (1.14)	2.66 (1.05)	0.002
	Post-intervention	2.91 (0.97)	2.83 (0.83)	0.249	2.82 (1.08)	2.87 (0.80)	0.512

[1] Independent sample *t*-test
